# AP-2 Transcription Factors as Regulators of Ferroptosis: A Family-Wide Profiling in Diverse Cancer Contexts

**DOI:** 10.3390/ijms27052310

**Published:** 2026-02-28

**Authors:** Damian Kołat, Piotr Gromek, Mateusz Kciuk, Lin-Yong Zhao, Żaneta Kałuzińska-Kołat, Renata Kontek, Elżbieta Płuciennik

**Affiliations:** 1 Department of Functional Genomics, Medical University of Lodz, 90-752 Lodz, Poland; piotr.gromek@stud.umed.lodz.pl (P.G.); mateusz.kciuk@biol.uni.lodz.pl (M.K.); zaneta.kaluzinska@umed.lodz.pl (Ż.K.-K.); elzbieta.pluciennik@umed.lodz.pl (E.P.); 2 Department of Molecular Biotechnology and Genetics, University of Lodz, 90-237 Lodz, Poland; renata.kontek@biol.uni.lodz.pl; 3 Department of General Surgery, West China Hospital, Sichuan University, Chengdu 610041, China; 153795352@scu.edu.cn; 4 Department of Biomedicine and Experimental Surgery, Medical University of Lodz, 90-136 Lodz, Poland

**Keywords:** ferroptosis, drivers, suppressors, markers, AP-2, *TFAP2A*, *TFAP2C*, *TFAP2E*, cell death regulators, pan-cancer

## Abstract

Ferroptosis is an iron-dependent programmed cell death (PCD) implicated in cancer therapy response, yet its transcriptional control remains unevenly characterized and often centered on a limited subset of transcription factors (TFs) rather than systematically addressing TF families. The Activating enhancer-binding Protein-2 (AP-2) family of TFs is a plausible but understudied regulatory node linking oncogenic programs to ferroptosis, with prior research limited to AP-2α and AP-2γ, suggesting anti-ferroptotic and pro-tumorigenic roles. Thus, the present study aimed to provide a family-wide analysis of the relationships between AP-2 and ferroptosis across tumors in which this PCD type is considered biologically and clinically relevant. The research integrates ferroptosis gene modules with AP-2 targetomes, tumor–normal expression comparisons, survival stratification, ferroptosis scoring, cross-cohort functional analyses, and signaling pathway projection extending canonical ferroptosis circuits with AP-2–associated non-canonical elements. Consistent associations between AP-2 expression, prognosis, and ferroptosis score were observed in five tumor cohorts: cervical squamous cell carcinoma, glioblastoma, ovarian serous cystadenocarcinoma, pancreatic adenocarcinoma, and thyroid carcinoma. In addition, cross-cohort clustering highlighted genes enriched in redox- and lipid-metabolism programs linked to apoptosis and autophagy-dependent death. Among the candidates emerging from these analyses, ferroptotic markers (*LOX*, *PTGS2*, and *NQO1*) and AP-2–linked nodes such as *CD36*, *DUOX1*, *EPHA2*, *MUC1*, *PTPRC*, *SNAI2*, and *TP63* warrant targeted functional and binding validation to infer whether these associations reflect direct AP-2 regulatory mechanisms. Most importantly, AP-2–centered research appears to be a valuable area for guiding studies of tumor-specific ferroptosis vulnerability or resistance.

## 1. Introduction

Programmed cell death (PCD) is a genetically regulated process by which cells actively execute their elimination in response to developmental cues or stress, thereby maintaining tissue homeostasis [1,2,3]. In contrast to uncontrolled cell death, PCD follows coordinated signaling cascades that enable efficient clearance of dying cells, limiting tissue damage and excessive inflammation [4]. Examples of PCD include apoptosis (characterized by chromatin condensation, DNA fragmentation, and apoptotic body formation) and necroptosis, a regulated necrotic program associated with membrane permeabilization and the release of cellular contents that can shape inflammatory signaling [4,5]. Autophagy, while often cytoprotective, can also contribute to regulated cell death through controlled cytoplasmic degradation pathways [3]. However, these programs must be tightly regulated to support proper development and immune regulation: insufficient PCD can allow the survival of damaged or transformed cells in cancer, while excessive or misdirected PCD increases the risk of degenerative pathologies [1,4]. Importantly, regulated cell death is managed by context-dependent programs in which transcription factors (TFs) integrate metabolic and stress cues to balance survival and elimination [6,7,8]. Beyond apoptosis, necroptosis, and autophagy-dependent death, a broader spectrum of PCD has been described, e.g., pyroptosis, ferroptosis, oxeiptosis, and alkaliptosis. This expansion has opened therapeutic opportunities for diseases such as cancer, as the pathways provide alternative routes to eliminate malfunctioning cells and may help overcome resistance to conventional therapies [3,9,10,11,12]. One of the most intensively studied processes within a broader PCD spectrum is ferroptosis, with substantial evidence supporting its relevance to tumor biology and therapeutic response [13,14].

Ferroptosis was originally defined as an iron-dependent, non-apoptotic form of regulated cell death, mechanistically distinct from apoptosis, necrosis, and autophagy [15]. In ferroptosis, iron availability enables Fe^2+^-catalyzed lipid peroxidation of polyunsaturated phospholipids through enzymatic and non-enzymatic mechanisms [16,17]. System Xc^−^, composed of solute carrier family 7 member 11 (SLC7A11) and solute carrier family 3 member 2 (SLC3A2), sustains glutathione (GSH) synthesis, while glutathione peroxidase 4 (GPX4) uses GSH to reduce lipid hydroperoxides [14,16]. Inhibition of either component, e.g., erastin targeting System Xc^−^ or RAS-selective lethal 3 (RSL3) targeting GPX4, depletes antioxidant capacity and lowers the ferroptotic threshold [18,19]. Beyond the canonical GPX4 axis, ferroptosis surveillance includes GPX4-independent defense nodes such as coenzyme Q10 (CoQ10) and ferroptosis suppressor protein 1 (FSP1). Notably, pharmacologic targeting of the latter can promote ferroptotic commitment via FSP1 phase separation and synergize with GPX4 inhibition [20]. Consistent with strong context dependence, the lymph node microenvironment has been reported to shift ferroptosis surveillance toward an FSP1 dependency linked to perinuclear lysosomes, highlighting lysosome biology as a relevant control point in vivo [21]. Iron loading is commonly discussed in terms of transferrin receptor-mediated uptake and ferritin-based storage/turnover (including nuclear receptor coactivator 4 (NCOA4)-mediated ferritinophagy), but emerging data emphasize that transferrin-independent routes can also increase redox-active intracellular iron [22,23]. In particular, endocytosis mediated by cluster of differentiation-44 (CD44) can serve as an alternative iron-acquisition mechanism in tumor contexts, expanding the iron-handling pathways relevant to ferroptosis [24]. Finally, recent chemical biology evidence supports a lysosome-centered framework in which ferroptotic lipid oxidation can be initiated in lysosomes: liproxstatin-1 was shown to exert cytoprotection by inactivating iron in lysosomes, GPX4 inhibition can initiate lipid oxidation within lysosomal membranes, and direct activation of lysosomal iron reactivity is sufficient to trigger ferroptosis in cancer models [25]. These lysosome-centered redox events are proposed to amplify lipid peroxidation beyond lysosomal membranes, promoting broader membrane damage and ferroptotic commitment. CD44 has become particularly relevant to modern ferroptosis models because it links cancer cell state (stem-like/mesenchymal programs) to iron endocytosis and redox-active iron availability. Mechanistically, CD44 can mediate the uptake of iron-bound hyaluronates, thereby increasing intracellular iron that can fuel lipid peroxidation [24]. More recently, membrane ether lipids were reported to enable CD44-dependent, non-clathrin-mediated iron endocytosis, increasing redox-active iron and modulating ferroptosis susceptibility in metastatic/stem-like carcinoma states [26]. Collectively, these findings broaden the iron sources relevant to ferroptosis beyond transferrin-centric models and motivate inclusion of CD44 when interpreting ferroptosis-relevant circuits. All of these features underpin therapeutic interest in ferroptosis induction or inhibition across modalities and in contexts of treatment sensitization and resistance [13,14].

As ferroptosis is determined by the balance between iron-handling components, antioxidant defenses, and lipid metabolic enzymes, its regulation is not only biochemical but also shaped by transcriptional programs that tune the death threshold in a context-dependent manner [16,27]. In PCD, cell fate can be influenced by the combined activity and timing of transcription factors that coordinate stress adaptation and elimination programs [6,28]. In ferroptosis, transcription factors modulate cellular sensitivity to canonical inducers (e.g., system Xc^−^ or GPX4 inhibitors) by adjusting the expression of iron transporters, antioxidant enzymes, and lipid metabolic genes, thereby controlling the threshold for ferroptotic death [29]. Within this regulatory landscape, Cap’n’collar (CNC) and CNC-related transcription factors, particularly NRF2, NRF1, and BACH1, have emerged as central regulators of ferroptosis [29,30]. NRF2 and NRF1 generally promote ferroptosis resistance by elevating antioxidant and iron-buffering programs, whereas BACH1 can act in a pro-ferroptotic manner by repressing genes involved in GSH synthesis and iron homeostasis to lower the threshold for ferroptosis induction [29,30]. Despite these advances, ferroptosis-focused transcriptional research remains uneven and often centers on a limited subset of TFs rather than a broader scope of transcription factor families. An underexplored regulatory node linking oncogenic transcriptional programs to ferroptosis control is the Activating enhancer-binding Protein-2 (AP-2) family of transcription factors [31,32]. The family comprises five TFs, viz. AP-2α, AP-2β, AP-2γ, AP-2δ, and AP-2ε (encoded by *TFAP2A-E* genes) that share conserved DNA-binding or dimerization features and bind GC-rich regulatory elements as homo- or heterodimers, supporting both potentially redundant and isoform-specific transcriptional outputs [33,34]. AP-2 proteins are established regulators of development and cancer phenotypes, including proliferation, epithelial–mesenchymal transition, stemness, and therapy resistance, making them strong candidates for controlling stress-response thresholds in malignant contexts [31].

To date, ferroptosis research on AP-2 has been limited to AP-2α and AP-2γ, revealing their pro-tumorigenic and anti-ferroptotic functions. AP-2α suppresses ferroptosis in gallbladder carcinoma by transcriptionally activating the NRF2 oxidative stress response axis, including *NFE2L2*, *HMOX1*, *NQO1*, and *FTH1* genes. When silenced, it elevates intracellular Fe^2+^ and malondialdehyde (a lipid peroxidation marker), reduces cell proliferation, migration, and invasion, and shifts gallbladder cancer cells toward ferroptotic vulnerability [31]. Similarly, AP-2γ inhibits ferroptosis by directly activating *GPX4*, *GPX1*, *EGFR*, and *YAP1/TAZ*, while simultaneously repressing *CDKN1A/p21*. These actions promote chemoresistance (e.g., to docetaxel in prostate and bladder cancer) and confer resistance to lipid peroxidation in breast, pancreatic, and bladder tumors [31,32]. The influence of remaining TFs in the AP-2 family (i.e., AP-2β, AP-2δ, and AP-2ε) on ferroptosis remains entirely uninvestigated. It is unusual considering their established roles in cancer progression: AP-2β has been associated with renal cell dedifferentiation and epithelial–mesenchymal transition, AP-2δ with prostate cancer aggressiveness, and AP-2ε with colorectal cancer chemoresistance [35,36,37]. Furthermore, the fact that AP-2α and AP-2γ are known to be involved in ferroptosis suggests that the remaining family members may also participate in ferroptosis regulation, either through overlapping transcriptional programs or through unique target gene networks that are yet to be characterized.

Therefore, the present study aims to provide the first family-wide analysis of the relationship between all AP-2 transcription factors and ferroptosis across tumors in which this PCD type has been established as a biologically and clinically relevant process. The study integrates ferroptotic genes with AP-2 targetomes and tumor transcriptomes to enable expression profiling, survival-linked stratification, and ferroptosis scoring. The findings indicate tumor contexts in which AP-2 expression is associated with ferroptosis potential; they also highlight cross-cohort candidate genes linking ferroptosis to other PCDs, biological processes, and signaling pathways. This workflow provides a resource for future studies on AP-2–centered regulatory networks as determinants of ferroptosis and related therapeutic response.

## 2. Results

### 2.1. Pan-Cancer Profiling Reaffirmed the Predominance of AP-2α/γ and Implicated AP-2ε in Ferroptotic Gene Modules, Highlighting Novel AP-2–Linked Ferroptosis Candidates

**Figure 1:**
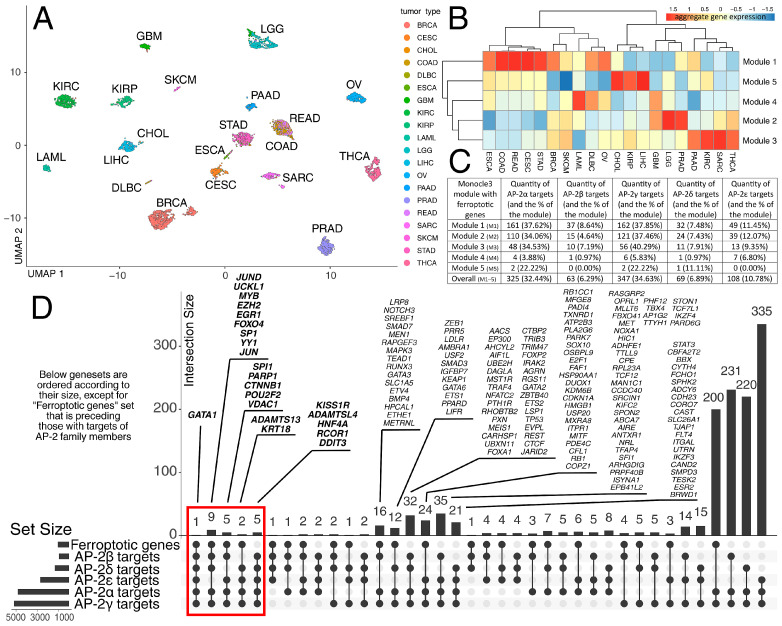
Pan-cancer profiling of ferroptotic genes alongside their intersection with targets of AP-2 transcription factors. (**A**) Expression profiling differences between tumors. (**B**) Subdivision of FPGs into modules with differential expression in cohorts. (**C**) The proportion of AP-2 targets across gene modules. (**D**) Intersection of AP-2 target genes with FPGs regardless of module clustering (red frame/rectangle indicates genes found in at least 5 intersected groups). Abbreviations in (**A**): Breast invasive carcinoma (BRCA); Cervical and endocervical cancers (CESC); Cholangiocarcinoma (CHOL); Colon adenocarcinoma (COAD); Diffuse large B-cell lymphoma (DLBC); Esophageal carcinoma (ESCA); Glioblastoma multiforme (GBM); Kidney renal clear cell carcinoma (KIRC); Kidney renal papillary cell carcinoma (KIRP); Acute myeloid leukemia (LAML); Lower-grade glioma (LGG); Liver hepatocellular carcinoma (LIHC); Ovarian serous cystadenocarcinoma (OV); Pancreatic adenocarcinoma (PAAD); Prostate adenocarcinoma (PRAD); Rectum adenocarcinoma (READ); Sarcoma (SARC); Skin cutaneous melanoma (SKCM); Stomach adenocarcinoma (STAD); Thyroid carcinoma (THCA).

Initially, the expression profiles of ferroptotic genes (FPGs) were visualized across 20 tumor cohorts (Figure 1A), which were identified as relevant for ferroptosis research (see Section 4.1 for details). Five gene modules showed distinct expression profiles across the studied tumor cohorts (Figure 1B). For instance, a particular profile for module 1 was observed in CESC and the four gastrointestinal tumors (ESCA, COAD, READ, STAD), while module 3 demonstrated similar expression in cohorts such as THCA, SARC, KIRC, and PAAD. The proportion of targets regulated by all AP-2 transcription factors was also assessed for all modules (Figure 1C). AP-2α and AP-2γ showed a clear predominance, followed by AP-2ε, while a similarly small number of targets were identified for AP-2β and AP-2δ. AP-2α/γ predominance likely reflects roles in epithelial cancer programs, whereas AP-2ε enrichment may indicate a more context-specific state with distinct stress-response constraints. In the first two modules, the contribution of targets was above 30% for AP-2α and AP-2γ, above 10% for AP-2ε, and below 10% for AP-2β and AP-2δ. Similar trends were observed in the third module, whereas the remaining two modules had the fewest targets for all AP-2 TFs. The AP-2 targets were also aligned with ferroptotic genes, regardless of module clustering (Figure 1D). This alignment identified FPGs that overlap with putative AP-2 target annotations in public resources, including genes annotated as targets of multiple AP-2 family members. There was also a visible intersection between targets of AP-2α/AP-2γ and two other studied sets (up to several dozen genes) or one other studied set (up to several hundred genes).

### 2.2. AP-2 Expression Was Linked to Tumor–Normal Differences and Cancer Patient Prognosis Across Multiple Cohorts, with Consistent Associations Noted in Ten Tumors

Prior to investigating the relationship between ferroptosis and AP-2 factors, the expression of each AP-2 family member was compared between tumor and normal tissues, and its impact on cancer patient prognosis was evaluated (Table 1). Of the 20 cohorts analyzed, AP-2 was found to influence patient survival in 14 tumors (Figure S1). However, the results of survival analysis were consistent with tumor vs. normal differences in 10 tumors. Of these, six cohorts (COAD, LGG, LIHC, OV, PAAD, THCA) were supported by at least two independent repositories containing tumor–normal data, and four cohorts (CESC, GBM, KIRC, SARC) by one such repository.

### 2.3. Consistent AP-2–Related Associations Revealed Ferroptosis Score Differences in Five Tumors

**Figure 2:**
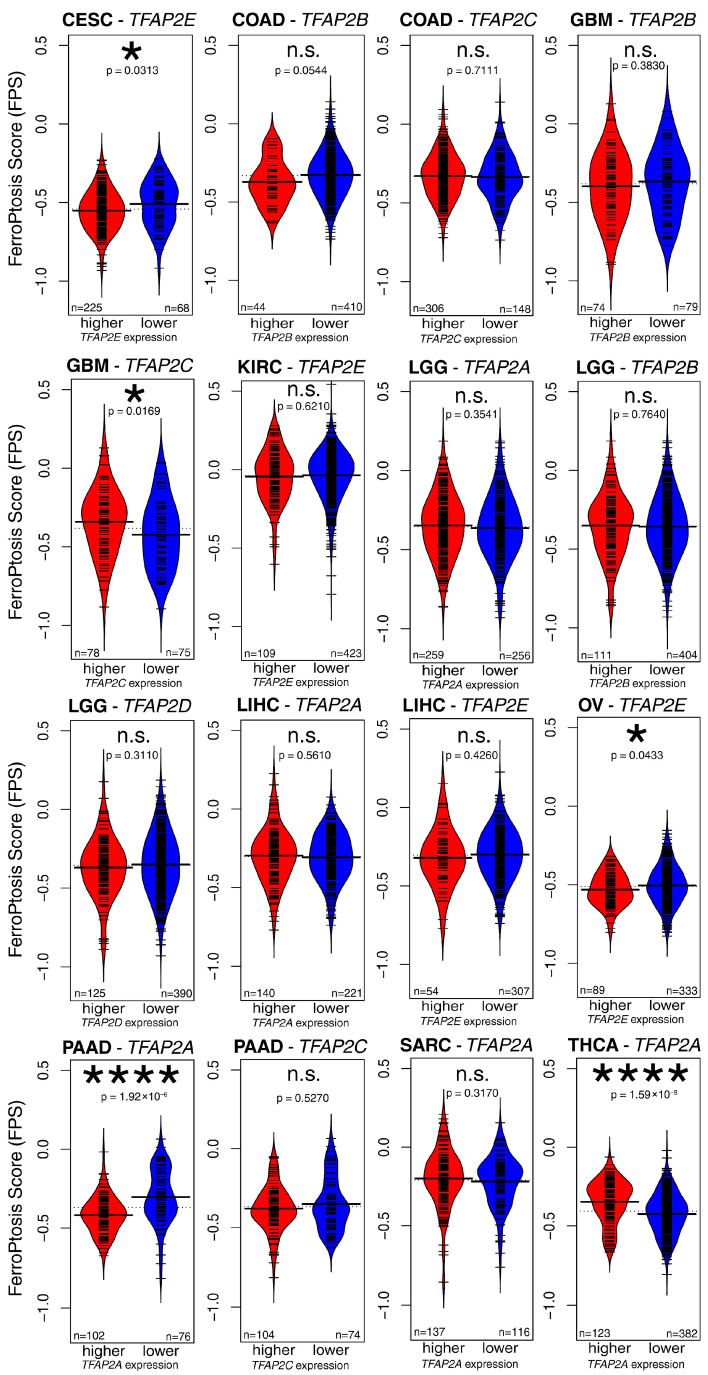
Assessment of ferroptosis score among survival analysis-derived groups with distinct expression of AP-2 family members. *p* > 0.05 (n.s.), *p* < 0.05 (*), *p* < 0.0001 (****).

Among the 10 tumors listed above, 16 observations showed that a specific AP-2 affected patient survival, which was also supported by differences in expression between tumor and normal tissue (Table 1). Groups were formed on the basis of survival analysis, and ferroptosis score (FPS) was determined for patients with distinct AP-2 levels (Figure 2). In five cases (*TFAP2E* in CESC, *TFAP2C* in GBM, *TFAP2E* in OV, *TFAP2A* in PAAD, and *TFAP2A* in THCA), groups with various AP-2 expression significantly differed in terms of FPS.

### 2.4. FPGs-Based Clustering of Cancer Patients Uncovered Cross-Cohort Discriminant Genes Linked to Ferroptosis, Apoptosis, Autophagy, and Diverse Biological Phenomena

**Figure 3:**
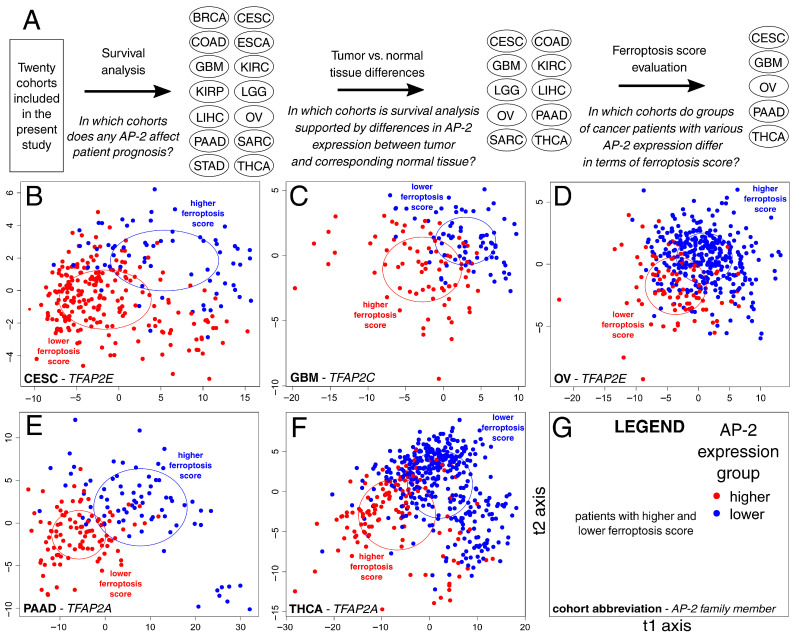
Patient clustering based on the expression profiles of ferroptotic genes in tumor cohorts with significant relationships between AP-2, survival, and ferroptosis score. (**A**) Flowchart explaining the selection of cohorts during specific study stages preceding patient clustering. (**B**) CESC. (**C**) GBM. (**D**) OV. (**E**) PAAD. (**F**) THCA. (**G**) Legend for (**B**–**F**) subfigures.

**Figure 4:**
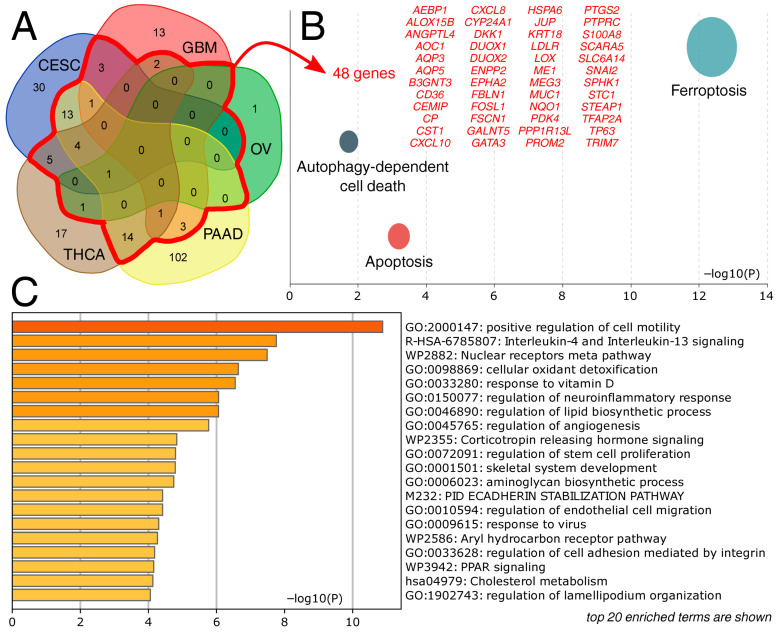
Functional annotation of discriminant genes. (**A**) Establishment of cross-cohort gene set. (**B**) Gene Ontology focused on cell death types. (**C**) Gene Ontology focused on biological processes and signaling pathways.

Patients from the five tumor cohorts with significant relationships between AP-2, survival, and FPS were spatially clustered by their FPG expression profile (Figure 3). The most important genes influencing this clustering in a given cohort were identified by discriminant analysis (see Section 4.6 for more details). These genes were compared across cohorts (Figure 4A), and the portion with any degree of overlap was functionally annotated. Apart from ferroptosis, overlapping genes were found to be involved in apoptosis and autophagy-dependent cell death (Figure 4B). They were also associated with various biological processes and signaling pathways, including cell motility, interleukin-4 and -13 signaling, detoxification, neuroinflammatory response, lipid biosynthesis, and angiogenesis (Figure 4C).

### 2.5. Cross-Cohort Genes Comprised Ferroptosis Drivers or Suppressors, Included Markers and AP-2 Targets, as Well as Showed Tumor-Specific Differential Expression and Prognostic Relevance

**Figure 5:**
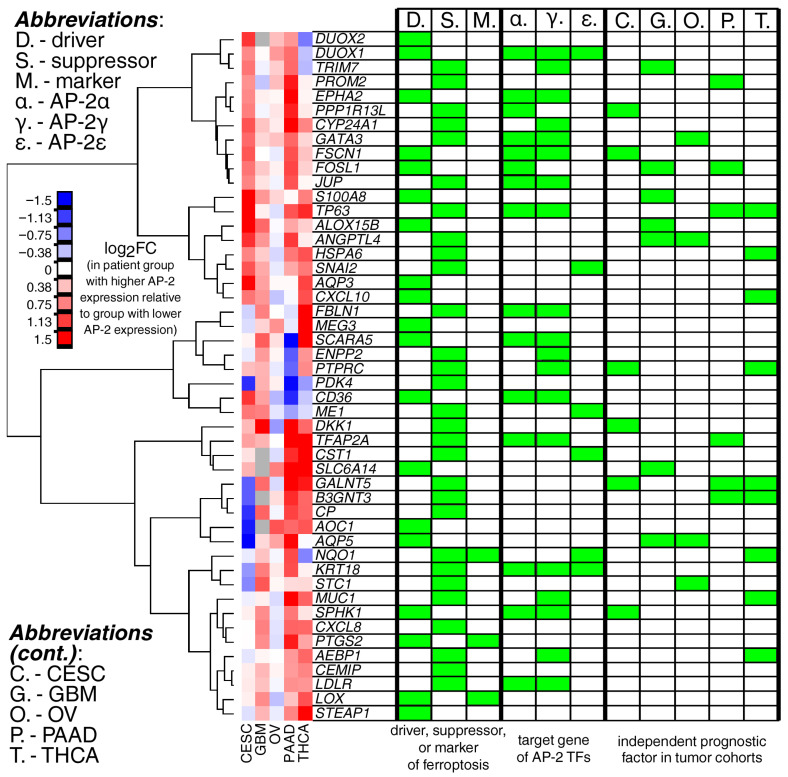
Differential expression analysis of cross-cohort genes, together with details on their role in ferroptosis, regulation by AP-2, and prognostic relevance. Abbreviations are explained in the graph. Green rectangles indicate that a given condition (described in the lower right corner) has been met.

The five cohorts were subjected to differential expression analysis of the cross-cohort genes identified in the previous stage (Figure 5); these genes were also characterized with respect to their roles in ferroptosis (Supplementary File S1), regulation by AP-2 (Supplementary File S2), and prognostic relevance (Supplementary File S3). The set contained 29 suppressors and 19 drivers of ferroptosis, and more than half of the studied genes were targets of at least one AP-2 transcription factor. Three of the genes were markers of ferroptosis, viz. *LOX*, *PTGS2*, and *NQO1*, with the latter also being a target for AP-2ε. The highest number of independent prognostic factors was observed in THCA, followed by GBM, CESC, PAAD, and OV. Several of the analyzed FPGs were of prognostic significance in at least two tumors; however, some of these genes were not found to be targets of any AP-2 TF (drivers: *AQP5*; suppressors: *ANGPTL4*, *B3GNT3*, *GALNT5*), or were targets of at least one TF (drivers: *FOSL1*; suppressors: *PTPRC*, *TP63*).

### 2.6. Integrating Selected Cross-Cohort Genes into the Ferroptosis Pathway Highlighted Complex Non-Canonical Signaling Linked to Apoptosis, Autophagy, and Broader Regulatory Pathways

**Figure 6:**
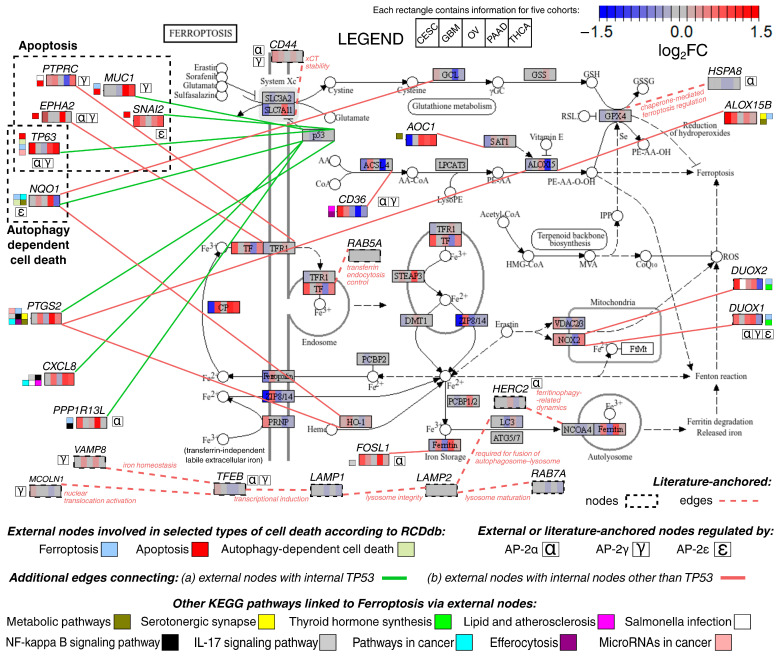
Ferroptosis pathway enriched with cross-cohort genes supported by interaction evidence and functional annotation (legend in the lower part of the graph). Data from differential expression analysis are given in rectangles (legend in the upper part of the graph). Dashed-outline nodes and edges indicate literature-anchored additions to include information on, e.g., lysosome-centered and CD44-linked iron uptake related to ferroptosis. Notes accompanying literature-anchored nodes explain the context of added value.

Ultimately, results of differential expression analysis (DEA) were also mapped to the canonical ferroptosis pathway (Figure 6). This signaling was enriched with data on those of the cross-cohort genes that: (a) were functionally annotated in the analysis focused on cell death types, (b) had confirmed protein–protein interactions (PPIs) with at least one representative of the canonical ferroptosis pathway, and (c) were included in other pathways of the Kyoto Encyclopedia of Genes and Genomes (only those pathways were selected if at least two genes from the analyzed list were identified). The outcome reflects the complexity of non-canonical ferroptosis signaling; it highlights its connections to apoptosis and autophagy-dependent cell death, as well as to other regulatory pathways, such as those associated with metabolism, microRNA, and efferocytosis. Among the externally visualized genes, most were AP-2 targets, viz. *CD36*, *DUOX1*, *EPHA2*, *FOSL1*, *MUC1*, *NQO1*, *PPP1R13L*, *PTPRC*, *SNAI2*, *TP63*. Many externally added nodes were associated with *TP53*; thus, distinct edge coloring was needed for other internal interactors, i.e., those encoded by the *ACSL1*, *ALOX15*, *CYBB*, *FTH1*, *FTL*, *GCLM*, *GCLC*, *HMOX1*, *SAT1*, *SAT2*, *SLC11A2, SLC40A1*, and *TFRC* genes. Pleiotropic functions were noted for some externally added nodes, particularly *TP63*, *CXCL8*, *PTGS2*, and *NQO1*; of these, the latter two were mentioned above as markers of ferroptosis, and the last one as a target of AP-2ε. Pathway projection was enriched with a set of literature-anchored nodes encompassing CD44 and those representing lysosome trafficking or chaperone-mediated regulation as determinants of redox-active iron and lipid peroxidation sensitivity.

## 3. Discussion

Ferroptosis and the AP-2 family have typically been investigated as independent topics, and the limited joint research has focused only on AP-2α (*TFAP2A*) and AP-2γ (*TFAP2C*). Through a multi-layered workflow, this study provides the first family-wide analysis across all AP-2 factors (*TFAP2A-E*), integrating dependent transcriptomic profiles with ferroptosis signatures across multiple tumors and selecting the most relevant relationships.

The module-level patterns and targetome overlaps support a model in which AP-2 TFs track distinct ferroptosis-related transcriptional states across tumors, rather than pointing to a single uniform AP-2/ferroptosis relationship. The predominance of target genes of AP-2α and AP-2γ likely reflects their established roles in epithelial tumor biology and differentiation programs [38,39]. AP-2ε is of particular interest, as up till now it has been linked mainly to gastrointestinal cancer drug resistance [34]. Its enrichment may mark a distinct tissue-restricted biology with potentially different redox and iron-handling constraints. The shared module-level profiles across CESC and some gastrointestinal cohorts suggest partially convergent ferroptosis-related transcriptional programs across these diseases. This observation is in line with previous findings: the gastrointestinal tumors ESCA, STAD, COAD, and READ were found to demonstrate similar ferroptosis gene expression and prognostic scores, while CESC and ESCA were stratified into comparable high and low ferroptosis classes in a multi-omics model based on TCGA data, suggesting transcriptomic overlap [40,41]. Interestingly, AP-2ε emerges as a novel candidate ferroptosis regulator alongside AP-2α and AP-2γ, with its targets enriching the most extensive gene modules that also contain ferroptosis markers *LOX*, *PTGS2*, and *NQO1*, the latter of which is an AP-2ε–specific target. Nevertheless, this does not establish it as a ferroptosis regulator in vivo, and focused investigation is still required. Previously, AP-2α was found to modulate ferroptosis in gallbladder carcinoma by activating the NRF2 pathway and increasing *NRF2*, *HO-1*, *NQO1*, and *FTH1* expression; its silencing elevated Fe^2+^ and malondialdehyde levels, shifting cells toward ferroptotic vulnerability [32]. Regarding AP-2γ, it induced *GPX4* expression to prevent lipid peroxidation, a crucial step in ferroptosis [42]. Knowledge on the roles of AP-2α and AP-2γ in ferroptosis is summarized elsewhere [31]. In contrast, AP-2β shows no direct impact on ferroptosis, which is consistent with the paucity of its targets in the gene modules identified herein. Literature data indicate it promotes thyroid cancer growth via COX-2 but without altering GPX4, lipid ROS, or sensitivity to erastin/RSL3 [43]. AP-2δ lacks ferroptosis associations, focusing on developmental transcription control according to the Universal Protein Knowledgebase (UniProtKB), GeneCards, and the National Center for Biotechnology Information (NCBI), and does not affect iron, ROS, or lipid peroxidation. Such a distinction may be related to its unique binding preferences [44].

Our alignment of FPGs with AP-2 targets reveals a promising gene set for further research, with *GATA1* showing connections to both ferroptosis and all AP-2 family transcription factors. *GATA1* promotes ferroptosis by upregulating *LMCD1*, driving Hippo-YAP and NRF2 degradation to amplify lipid peroxidation in tumor cells under stress [45]. Its appearance in the lists of AP-2 targets, together with *GATA2*, may imply broader GATA switching: in cancer differentiation, GATA1 displaces GATA2 at shared motifs through FOG-1/NuRD chromatin repression, shifting from tumor progenitor maintenance to ferroptosis vulnerability [46]. GATA2 has been found to partner with AP-2α/γ and GATA3 to regulate stemness and epithelial-to-mesenchymal transition (EMT) in cancer stem-like cells, which may be associated with ferroptosis evasion in aggressive malignancies [46,47]. Another noteworthy gene showing connections to ferroptosis and most AP-2 TFs is *EZH2*. The gene encodes the H3K27me3 methyltransferase, a catalytic subunit of the polycomb repressive complex 2, which suppresses ferroptosis by epigenetically silencing TFR2, limiting iron import and lipid peroxidation, and enhancing sorafenib resistance [48,49]. AP-2α activates *EZH2* via the E2F pathway in melanoma, driving metastasis through epigenetic repression [48]. The presence of *EZH2* is associated with an unfavorable prognosis [49] and appears to serve as a bridge between ferroptosis and AP-2 in cancer. Hence, it is possible for AP-2α to inhibit ferroptosis in a similar way to AP-2γ [31].

Across multiple cohorts, AP-2 expression showed significant survival associations that aligned with tumor–normal deregulation, supporting the clinical relevance of AP-2–linked transcriptional states in ferroptosis-relevant tumors. *TFAP2A* upregulation in tumors relative to normal tissues was associated with an unfavorable prognosis in some of these cohorts [50]. Other AP-2 family members also show cohort-specific deregulation with prognostic relevance, e.g., *TFAP2C* upregulation in colorectal tumors with worse survival, as well as *TFAP2E* promoter methylation or *TFAP2B* overexpression in colorectal or thyroid cancer, respectively [43,51,52]. In the five focal cohorts (CESC, GBM, OV, PAAD, THCA), survival-linked AP-2 stratification coincided with significant shifts in the ferroptosis score, suggesting that AP-2 status tracks ferroptosis-related transcriptional potential. This represents a novel finding, as among these tumors, ferroptosis regulation has previously been demonstrated only for *TFAP2A* in pancreatic cancer. In addition, *TFAP2C* expression has been linked to ferroptosis in other cohorts, including breast cancer [53,54]. Hence, AP-2ε represents a novel, important TF in ferroptosis modulation, a role previously attributed only to AP-2α and AP-2γ, which typically exert a suppressive influence on this PCD type. Across cohorts, the association between AP-2 expression and FPS was context-dependent, with higher AP-2 aligning with either lower or higher FPS depending on tumor type. As FPS reflects transcriptomic ferroptosis potential rather than execution, the presence of elevated FPS alongside high AP-2 may indicate adaptive antioxidant engagement, which can still be associated with worse outcomes.

The cross-cohort discriminant set points to ferroptosis being embedded within broader apoptosis- and autophagy-linked stress programs, consistent with tumor state adaptation rather than a ferroptosis-only axis. The annotated processes and pathways are coupled to ferroptotic execution or resistance. Firstly, canonical ferroptosis-suppressive systems such as glutathione/GPX4 and FSP1/CoQ10 axes, as well as NRF2-regulated antioxidant enzymes, play roles in cellular oxidant detoxification; all raise the threshold for ferroptotic death by limiting lipid ROS accumulation. This suggests that AP-2 may modulate ferroptosis by influencing detoxification capacity in these tumors, which is consistent with *TFAP2A*-driven activation of the NRF2 axis described in other contexts [55]. Consistent with microenvironment-dependent ferroptosis surveillance, lymph node metastasis has been associated with increased FSP1 dependence and perinuclear lysosome localization, reinforcing lysosome biology as a relevant context when interpreting ferroptosis-related programs [21]. Secondly, enrichment in the regulation of lipid biosynthetic processes and cholesterol metabolism was observed, indicating that, to some extent, AP-2 controlled the availability and composition of the lipid substrates that determine ferroptosis sensitivity. Polyunsaturated phospholipids generated via *ACSL4/LPCAT3*-dependent signaling are essential substrates for peroxidation, while mevalonate/cholesterol metabolism supplies isoprenoids and CoQ10 that feed into FSP1-CoQ10 and GPX4 selenocysteine synthesis, collectively buffering lipid peroxidation [55,56]. Finally, the enrichment of peroxisome proliferator-activated receptor (PPAR) signaling reflects the integration of transcriptional control of lipid metabolism with redox homeostasis: PPARs coordinate fatty-acid metabolism and antioxidant gene expression, which modulate susceptibility to lipid peroxidation in hepatic and cancer models [56]. The presence of PPAR-linked networks in our ontology suggests a layered regulatory architecture whose activity is fine-tuned by AP-2 TFs. As such, further investigation into CESC, GBM, OV, PAAD, and THCA is needed in this regard.

Pathway projection of discriminant genes onto the canonical ferroptosis signaling highlighted pleiotropic nodes that may connect AP-2–associated states with redox defense and inflammatory signaling. Notably, *NQO1* emerged as one such node, serving as both a ferroptosis marker and an AP-2ε target. *NQO1* encodes a flavoprotein that catalyzes the two-electron reduction of quinones to hydroquinones and serves as a component of the cellular ferroptosis defense machinery; this is consistent with its role in limiting oxidative stress and lipid peroxidation [57,58]. As a pan-cancer prognostic biomarker, its overexpression consistently predicts poor overall survival in lung, colon, liver, breast, pancreatic, and thyroid cancers [59,60]. While early evidence confirmed that AP-2 binding to the *NQO1* promoter is involved in basal/cAMP-mediated regulation [61], the mechanistic crosstalk remains unexplored. NQO1-bioactivatable quinones, such as β-lapachone, can drive strong ROS bursts via redox cycling and have been linked to ferroptotic phenotypes; thus, they exhibit therapeutic tunability. The non-canonical ferroptosis axis, potentially incorporating AP-2ε, NQO1, and ROS, appears yet to be fully established and utilized. Furthermore, some EMT/stemness regulators in our AP-2–linked gene set have established roles in ferroptosis: for example, TP63–GPX4 signaling confers resistance [62], SNAI2 promotes sensitivity [63], and CD36-mediated lipid uptake modulates cell death [64]. Therapy-resistant mesenchymal states can exhibit a heightened dependence on GPX4 and thus vulnerability to ferroptotic triggers [65,66]. Conversely, cancer stem-like CD44 variants can stabilize the xCT antiporter, sustain glutathione levels, and buffer oxidative stress, providing a route to ferroptosis evasion [67]. As CD44-dependent iron endocytosis has been shown to increase intracellular redox-active iron in tumor contexts [24,26], CD44 may function as an iron-loading node that intersects with both stemness and ferroptosis outcomes. Because CD44-mediated transferrin-independent iron uptake is not represented in the canonical KEGG ferroptosis map, we annotated CD44 in pathway projection as a literature-supported iron-uptake node. Overall, as some AP-2 family members are established regulators of epithelial differentiation and luminal lineage programs [31,38,39], their overexpression may drive a mesenchymal/stem-like phenotype that elevates iron buffering and lipid remodeling, raising the ferroptosis threshold.

The study has several limitations that should be acknowledged. Because our analyses rely on publicly available bulk RNA-Seq and intersections with AP-2 targetome resources, the reported AP-2–ferroptosis relationships do not establish direct binding or causal directionality, necessitating AP-2 perturbation combined with TF binding assays (e.g., ChIP-Seq/CUT&RUN/CUT&Tag). In addition, FPS is an expression-signature proxy that captures regulatory potential rather than ferroptotic execution. Therefore, higher FPS in AP-2-high tumors could also reflect compensatory antioxidant or iron-buffering programs rather than increased ferroptotic cell death in vivo. Accordingly, one should not equate FPS associations with direct ferroptosis occurrence without orthogonal biochemical and functional validation (e.g., lipid peroxidation assays and rescue by ferroptosis inhibitors). Moreover, bulk transcriptomes are sensitive to tumor purity and immune/stromal admixture. Thus, correlations involving immune and inflammatory markers, such as *PTPRC* or *CXCL8*, may reflect compositional differences rather than tumor-intrinsic regulation. These constraints are well recognized in bulk tumor transcriptomics and motivate future assessment using cell-type deconvolution and spatial transcriptomic approaches. Finally, AP-2 targetome-based inferences are limited by the availability of public binding/target annotations for some family members.

In summary, this study provides a family-wide map linking AP-2 transcription factors to ferroptosis-related expression programs and clinical outcomes, highlighting cohort-specific AP-2/FPS phenotypes and indicating that AP-2ε merits further investigation alongside AP-2α and AP-2γ. Priority areas for future research include: (1) mechanistic validation of AP-2–dependent ferroptosis modulation (genetic perturbation with lipid-ROS/iron readouts and sensitivity to ferroptosis inducers); (2) resolving cell-type specificity using single-cell approaches and protein-level validation; (3) testing whether AP-2 status predicts therapy response or enables rational combination strategies; and (4) extending the ferroptosis pathway beyond canonical genes to the networks connecting redox metabolism with apoptosis and autophagy-dependent death. The potential for AP-2 family members or their targets to serve as biomarkers and therapeutic candidates in ferroptosis-based strategies warrants further investigation. Combining AP-2 status with ferroptotic readouts at the RNA and protein levels may help guide the use of redox-modulating drugs and support the selection of combination therapies better tailored to tumor-specific ferroptosis programs.

## 4. Materials and Methods

### 4.1. Tumor Cohort Selection and Transcriptomic Data Acquisition

Suitable tumors for ferroptosis research were identified using the Ferroptosis Database v2 (FerrDb v2; Zhou Lab, Guangzhou Medical University, Guangzhou, China) [55] (only records with status “validated” for “human” were included), supported by a literature data summarizing clinical trial findings [68]. The obtained data were matched to cohorts in The Cancer Genome Atlas (TCGA); as a result, 20 tumors were included in the study (Table 2). Transcriptomic data for these diseases were obtained from the Genomic Data Commons (GDC) repository [69] (accessed on 13 August 2025) using the GDCquery() function in the TCGAbiolinks v2.34.1 R-package (R Foundation for Statistical Computing, Vienna, Austria).

Data from each cohort were restricted to the RNA-Seq experimental strategy, with one primary tumor sample retained per patient, prioritizing the “A” vial (or another one if the “A” vial was missing). Normal tissue and metastatic samples were excluded. Transcriptomic data were downloaded as raw counts (Spliced Transcripts Alignment to a Reference protocol; STAR) and as normalized values (transcripts per million; TPM), with the 38th major build of the human genome by the Genome Reference Consortium (GRCh38/hg38) employed as the reference.

### 4.2. Establishment of Ferroptotic Gene Set and Lists of AP-2 Targets

Data for ferroptosis drivers, suppressors, and markers from the FerrDb v2 repository [70] were de-duplicated, filtered to exclude ambiguous records, and aligned with available expression data for the TCGA cohorts. Collectively, 422 drivers, 585 suppressors, and 57 markers were included in the analysis, with some markers belonging to the driver or suppressor group (Supplementary File S1). This set was denoted as ferroptotic genes (FPGs). Lists of targets for all AP-2 family members were obtained as per our previous research [71,72,73]. The following quantities of targets were investigated for each AP-2 representative: 4810 for AP-2α, 928 for AP-2β, 5175 for AP-2γ, 1318 for AP-2δ, and 2700 for AP-2ε (Supplementary File S2).

### 4.3. Evaluation of Ferroptosis Expression Patterns, Detection of Gene Modules, Enrichment of AP-2 Targets, and Intersection Analysis

The expression patterns of FPGs across the included tumor cohorts were evaluated using the Monocle3 toolkit (University of Washington, Seattle, WA, USA) [74]. Genes with no detectable expression across the analyzed samples were removed prior to subsequent steps. Pre-processing was performed using the preprocess_cds() function with the num_dim parameter set to 100. Dimensionality reduction was performed with the reduce_dimension() function, and cells were clustered with the cluster_cells() function, both using the Uniform Manifold Approximation and Projection (UMAP) algorithm as the base clustering method (reduction_method). Statistical validation was conducted using Moran’s I spatial autocorrelation with the graph_test() function and a kNN neighbor graph (q-value threshold 0.05). Genes were grouped into modules using the Louvain community detection algorithm via the find_gene_modules() function (k = 4) to identify clusters of co-expressed FPGs across tumors, which were further visualized with Ward D2 hierarchical clustering in pheatmap(). All analyses adhered to the official Monocle3 guidelines (https://cole-trapnell-lab.github.io/monocle3, accessed on 22 August 2025). Afterwards, the proportion of targets regulated by all AP-2 transcription factors in each Monocle3 module was assessed using the base v4.4.3 R-package (R Foundation for Statistical Computing, Vienna, Austria). Regardless of module clustering, an intersection analysis of AP-2 targets and FPGs was performed using the UpSetR v1.4.0 R-package (R Foundation for Statistical Computing, Vienna, Austria).

### 4.4. Multi-Platform Analysis of AP-2 Expression Differences Between Tumor Versus Normal Tissues and Prognostic Stratification of Cancer Patients

A multi-platform approach was applied to compare the expression of all AP-2 family members between tumor and normal tissues and to evaluate their impact on cancer patient prognosis. The Gene Expression database of Normal and Tumor tissues-2 (GENT2; (Korea Research Institute of Bioscience and Biotechnology, Daejeon, Republic of Korea) [75] was employed to assess AP-2 patterns using the “Search—Gene Profile” workflow. These patterns were also explored using the “Single Gene Analysis” function in the Gene Expression Profiling Interactive Analysis-2 (GEPIA2) database [76], as well as utilizing the search bar in the Oncopression repository [77], by inputting the relevant gene symbol (*TFAP2A*, *TFAP2B*, *TFAP2C*, *TFAP2D*, or *TFAP2E*). Using the EvaluateCutpoints v1 tool (Medical University of Lodz, Lodz, Poland) [78], the optimal expression cutpoint for each AP-2 family member was determined to stratify patients into two groups based on the disease-specific survival (DSS) endpoint (obtained from the TCGA Clinical Data Resource [79]; included samples summarized in Supplementary File S4). Corresponding Kaplan–Meier curves were visualized with the ggsurvplot() function from the survminer v0.5.0 R-package. The resulting patient groups, defined by AP-2 expression and DSS outcome, were used in downstream analyses.

### 4.5. Calculation of Ferroptosis Scores and Comparison Between Groups of Cancer Patients

Sample-level ferroptosis scores were calculated using the FPSOmics v0.1.0 (Shanghai Institute of Immunology, Shanghai, China) [80], which implements a published ferroptosis gene signature to compute each sample’s ferroptosis potential score from its expression data. The internal pro-ferroptosis and anti-ferroptosis signatures of the toolkit were evaluated on a gene-by-sample expression matrix. Enrichment was computed using the built-in Gene Set Variation Analysis (GSVA) with the Single Sample Gene Set Enrichment Analysis (ssGSEA) method (default settings), yielding enrichment scores for each sample. Ferroptosis scores were compared between the patient groups established during the survival analysis; normality of distribution was determined by the Shapiro–Wilk test, followed by an unpaired *t*-test or a Wilcoxon test.

### 4.6. Discriminant Analysis with Detection of Cross-Cohort Ferroptotic Genes and Their Functional Annotation

Genes with varying expression between samples, characterized by higher or lower FPS, were intersected across tumors and subjected to functional annotation. Within each tumor cohort, gene expression matrices were log2-transformed where required, zero-variance genes were removed, and features were z-scaled. Partial Least Squares Discriminant Analysis (PLS-DA) was performed using the ropls v1.38 R-package (R Foundation for Statistical Computing, Vienna, Austria); the procedure employed two predictive components and no orthogonal components. All genes found to be significant (*p* < 0.05) for the AP-2 higher-vs-lower contrast (established during survival analysis) were used as the feature set for supervised modeling. Samples were plotted on the t1 and t2 axes, with group dispersion indicated using Euclidean ellipses. Cross-cohort candidates were defined as genes present in at least two cohorts, and their overlap was visualized in a Venn diagram using the draw.pairwise.venn() function from the VennDiagram v1.7.3 R-package. Genes were functionally annotated using the Regulated Cell Death database (RCDdb) [81] to identify enriched cell death types, and utilizing Metascape [82] to infer involved biological processes and signaling pathways. Both databases were run with default settings, with RCDdb via the “Analysis” tab and Metascape via the “Express Analysis” functionality.

### 4.7. Differential Expression Analysis with Downstream Visualization and Prognostic Assessment of Cross-Cohort Genes

Differential expression analysis was conducted using the limma-voom workflow (limma v3.62.2 R package) [83,84] to find genes significantly up- and down-regulated between groups. The pipeline included normalization with the calcNormFactors() function and filtering for lowly expressed transcripts (≥5 counts per million across ≥ 1 libraries were retained), followed by variance modeling using the voom() transformation. The model was fitted in limma using weighted least squares for each gene via lmFit(), and log2FC values for the case group (higher expression of a given AP-2 family member) were compared to the control group (lower expression of a given AP-2 family member) using the makeContrasts() function with default settings. Empirical Bayesian moderation of standard errors preceded the retrieval of differentially expressed genes (*p* < 0.05 and log2FC > 0.58 or <−0.58) via the topTable () function. During some stages of the present study (as visualized in Section 2.5 and Section 2.6), genes not classified as DEGs were used as a background set. For the cross-cohort genes identified in the PLS-DA workflow, a heatmap was generated using the Next-Generation Clustered Heat Map (NG-CHM) v2.24.4 tool [85] with Ward agglomeration and the Euclidean distance metric for row clustering. The same gene set was evaluated using multivariate survival analysis via the Tumor online Prognostic analyses Platform (ToPP) [86] to determine independent prognostic factors. The data type was set to “gene expression”, the cutoff to “best”, and the survival type to “DSS”.

### 4.8. Ferroptosis Pathway Visualization and External Data Integration

To contextualize AP-2–associated patterns within the ferroptosis pathway, DEA results were mapped to the KEGG pathway representing ferroptosis (hsa04216) using the pathview v1.46 R-package (R Foundation for Statistical Computing, Vienna, Austria), with a bins parameter of 20 and a limit value of 1.5 for gene data when converting to pseudocolors. The KEGG native graph was enriched with additional data from the literature and the cross-cohort gene set: protein–protein interactions between this set and representatives of the ferroptosis KEGG pathway were assessed using the Search Tool for the Retrieval of Interacting Genes/Proteins (STRING) v12 (Swiss Institute of Bioinformatics, Lausanne, Switzerland) [87], which was set to output high-confidence results. Moreover, the KEGG repository (Kanehisa Laboratories, Kyoto University, Uji, Kyoto, Japan) [88] was used to identify pathways containing at least two cross-cohort genes. Afterwards, external data were integrated into the pathview-generated graph using Inkscape v1.4.2 (Inkscape Project; Software Freedom Conservancy, Brooklyn, NY, USA) [89].

## 5. Conclusions

This study provides a family-wide map of AP-2 transcription factors in the context of ferroptosis across twenty TCGA cohorts. Integration of FPG modules with AP-2 targetomes, tumor–normal expression patterns alongside AP-2–related survival stratification, ferroptosis scoring, cross-cohort functional investigation, and projection of candidate genes onto the ferroptosis pathway contextualizes AP-2–associated patterns without implying direct regulation. The findings highlight AP-2α and AP-2γ as the predominant AP-2 members within ferroptosis-linked transcriptional networks and propose AP-2ε as an additional candidate connected to this PCD type. Relationships between AP-2 expression, patient survival, and ferroptosis score were identified in five tumor cohorts: CESC, GBM, OV, PAAD, and THCA. Cross-cohort clustering identified discriminant genes enriched in redox homeostasis and lipid metabolism, with links to apoptosis and autophagy-dependent cell death, supporting the expansion of canonical ferroptosis via non-canonical signaling related to AP-2. Among the candidates emerging from these analyses, ferroptotic markers (*LOX*, *PTGS2*, and *NQO1*) and AP-2–linked nodes such as *CD36*, *DUOX1*, *EPHA2*, *MUC1*, *PTPRC*, *SNAI2*, and *TP63* warrant targeted functional and binding validation, especially *NQO1* due to its pleiotropy and relationship with AP-2ε. This study provides a resource for further AP-2–centered research aimed at defining ferroptosis vulnerability or resistance for specific tumors. Future studies should focus on genetic perturbation experiments with lipid peroxidation/iron readouts, single-cell and protein-level validation, contexts shaped by transferrin-independent iron uptake and lysosomal regulation, and on evaluating whether AP-2 status can aid patient stratification for ferroptosis-oriented therapeutic combinations.

## Figures and Tables

**Table 1:** Differences in AP-2 family expression between tumor and normal tissues, along with their associations with cancer patient prognosis.

Tumor Type	*TFAP2A*				*TFAP2B*				*TFAP2C*				*TFAP2D*				*TFAP2E*			
	GT2	GP2	O	EC	GT2	GP2	O	EC	GT2	GP2	O	EC	GT2	GP2	O	EC	GT2	GP2	O	EC
BRCA	↑	n.s.	↑	n.s.	↑	n.s.	↓	UF	↓	n.s.	n.s.	n.s.	↓	n.s.	↓	n.s.	↓	n.s.	n.s.	n.s.
CESC	↑	↑	n.d.	n.s.	n.s.	n.s.	n.d.	n.s.	n.s.	n.s.	n.d.	F	↓	n.s.	n.d.	n.s.	↓	n.s.	n.d.	F
CHOL	↑	n.s.	n.d.	n.s.	n.s.	n.s.	n.d.	n.s.	n.s.	n.s.	n.d.	n.s.	n.s.	n.s.	n.d.	n.s.	n.s.	n.s.	n.d.	n.s.
COAD	↑	n.s.	↑	n.s.	n.s.	n.s.	↑	UF	↑	n.s.	↑	UF	n.s.	n.s.	↑	n.s.	↓	n.s.	↑	n.s.
DLBC	n.s.	n.s.	n.d.	n.s.	n.s.	n.s.	n.d.	n.s.	n.s.	n.s.	n.d.	n.s.	n.s.	n.s.	n.d.	n.s.	n.s.	n.s.	n.d.	n.s.
ESCA	n.s.	n.s.	n.d.	F	↓	↓	n.d.	n.s.	n.s.	n.s.	n.d.	n.s.	n.s.	n.s.	n.d.	n.s.	n.s.	n.s.	n.d.	F
GBM	↑	↑	↑	n.s.	n.s.	n.s.	↑	UF	n.s.	n.s.	↑	UF	↓	n.s.	n.s.	n.s.	↓	↓	↑	F
KIRC	↓	↓	↓	UF	↓	↓	↓	n.s.	↓	n.s.	↑	UF	n.s.	n.s.	↓	UF	n.s.	n.s.	↑	UF
KIRP	↓	↓	↓	UF	↓	↓	↓	UF	↓	n.s.	↑	n.s.	n.s.	n.s.	↓	n.s.	n.s.	n.s.	↑	n.s.
LAML	↑	↓	n.d.	n.s.	↑	n.s.	n.d.	n.s.	↑	n.s.	n.d.	n.s.	↑	n.s.	n.d.	n.s.	↑	↑	n.d.	n.s.
LGG	↑	↑	↑	UF	n.s.	n.s.	↑	UF	n.s.	n.s.	↑	n.s.	↓	n.s.	n.s.	F	↓	↓	↑	F
LIHC	↑	↑	↑	UF	n.s.	n.s.	↑	n.s.	↑	↑	↑	n.s.	n.s.	n.s.	n.s.	n.s.	↑	n.s.	↑	UF
OV	↑	↑	↑	n.s.	n.s.	n.s.	↑	n.s.	↑	↑	↑	n.s.	n.s.	n.s.	↑	n.s.	n.s.	↓	↓	F
PAAD	↑	↑	↑	UF	n.s.	n.s.	↑	n.s.	↑	n.s.	↑	UF	n.s.	n.s.	↓	n.s.	n.s.	n.s.	n.s.	n.s.
PRAD	↑	n.s.	↑	n.s.	↑	n.s.	↑	n.s.	↑	n.s.	↑	n.s.	n.s.	n.s.	n.s.	n.s.	↓	n.s.	n.s.	n.s.
READ	↑	n.s.	↑	n.s.	n.s.	n.s.	↑	n.s.	↑	n.s.	↑	n.s.	n.s.	n.s.	↑	n.s.	↓	n.s.	↑	n.s.
SARC	n.s.	n.s.	↑	UF	n.s.	n.s.	↑	n.s.	n.s.	n.s.	↑	n.s.	n.s.	n.s.	↑	n.s.	n.s.	n.s.	↑	n.s.
SKCM	↑	n.s.	n.s.	n.s.	↑	↓	↓	n.s.	↓	↓	↓	n.s.	↑	n.s.	n.s.	n.s.	n.s.	↓	↓	n.s.
STAD	n.s.	↑	↑	F	↓	n.s.	n.s.	UF	n.s.	n.s.	↑	n.s.	↓	n.s.	↓	n.s.	↓	n.s.	n.s.	UF
THCA	↑	n.s.	↑	UF	n.s.	n.s.	n.s.	n.s.	n.s.	n.s.	↑	n.s.	n.s.	n.s.	n.s.	n.s.	n.s.	n.s.	n.s.	n.s.

GT2—GENT2; GP2—GEPIA2; O—Oncopression; EC—EvaluateCutpoints; n.s.—not significant; n.d.—no data; ↑—higher expression in tumor relative to normal tissue (marked with a light-red background color); ↓—lower expression in tumor relative to normal tissue (marked with a light-blue background color); UF—higher expression in tumor is unfavorable for prognosis (marked with a light-red background color); F—higher expression in tumor is favorable for prognosis (marked with a light-blue background color).

**Table 2:** The tumors included in the study, along with the corresponding cohort sizes.

TCGA Cohort Abbreviation	Full Disease Name/Description	Number of Samples Included in This Study *
BRCA	Breast invasive carcinoma	1095
CESC	Cervical and endocervical cancers	304
CHOL	Cholangiocarcinoma	35
COAD	Colon adenocarcinoma	458
DLBC	Diffuse large B-cell lymphoma	48
ESCA	Esophageal carcinoma	184
GBM	Glioblastoma multiforme	288
KIRC	Kidney renal clear cell carcinoma	533
KIRP	Kidney renal papillary cell carcinoma	290
LAML	Acute myeloid leukemia	151
LGG	Lower-grade glioma	516
LIHC	Liver hepatocellular carcinoma	371
OV	Ovarian serous cystadenocarcinoma	422
PAAD	Pancreatic adenocarcinoma	178
PRAD	Prostate adenocarcinoma	497
READ	Rectum adenocarcinoma	166
SARC	Sarcoma	259
SKCM	Skin cutaneous melanoma	103
STAD	Stomach adenocarcinoma	412
THCA	Thyroid carcinoma	505

* Group sizes reflect patients with available RNA-Seq data, not total project cases shown on the GDC repository. Cohorts were subjected to filtering: normal/metastatic tissue samples were excluded, and one primary tumor sample per patient was retained.

## Data Availability

The original contributions presented in this study are included in the article/Supplementary Materials. Further inquiries can be directed to the corresponding author.

## Supplementary Materials

The following supporting information can be downloaded at: https://www.mdpi.com/article/10.3390/ijms27052310/s1.
