# Supplementary figures and images for: AP-2 Transcription Factors as Regulators of Ferroptosis: A Family-Wide Profiling in Diverse Cancer Contexts
Source: Int J Mol Sci. 2026 Feb 28;27(5):2310. doi: 10.3390/ijms27052310 (PMC12986138; doi:10.3390/ijms27052310)

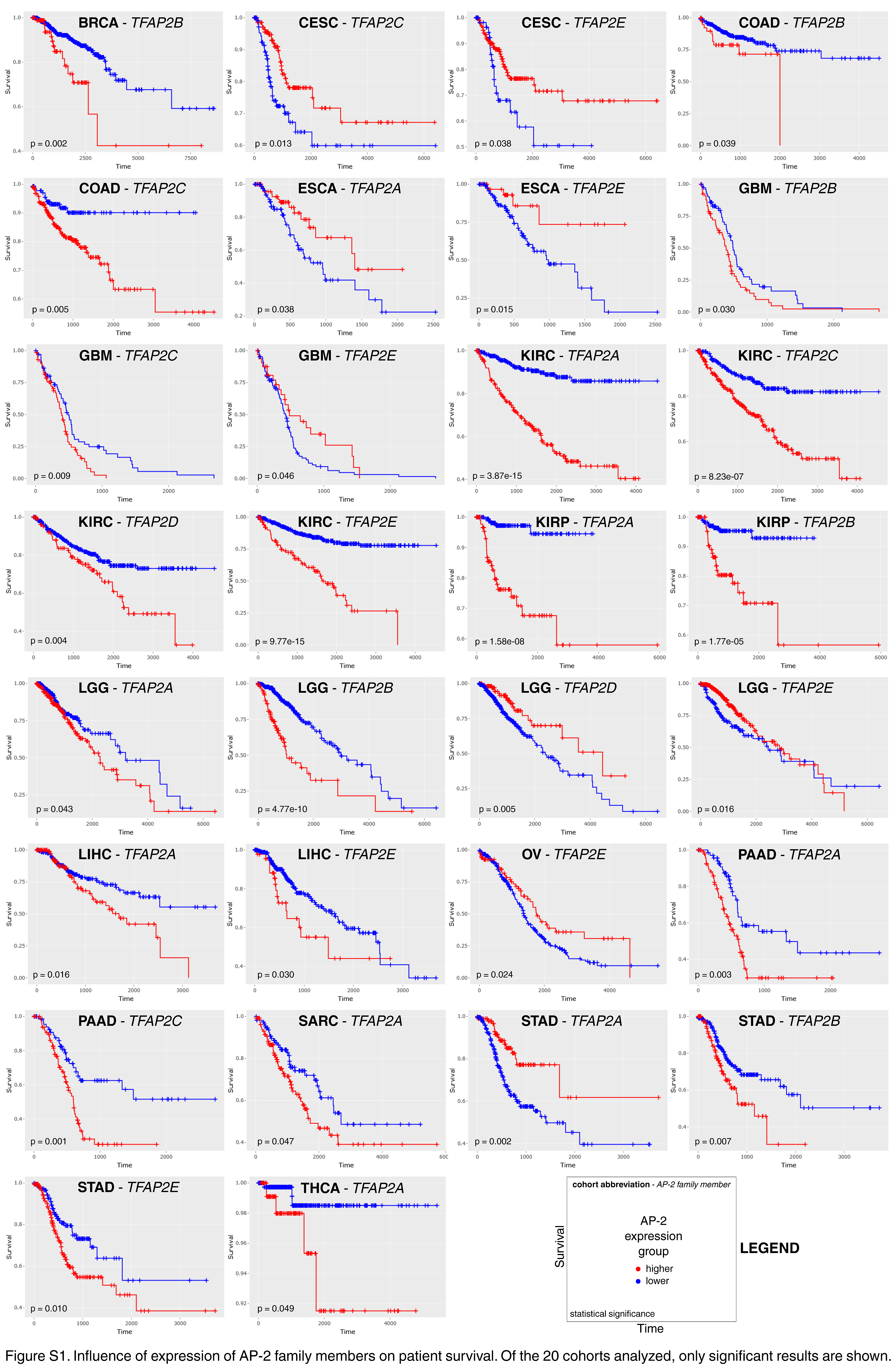

Supplement: Supplementary file 1 [file ijms-27-02310-s001.zip › Supplementary_materials/Figure_S1.tif]
